# Combination of Acupoints in Treating Patients with Chronic Obstructive Pulmonary Disease: An Apriori Algorithm-Based Association Rule Analysis

**DOI:** 10.1155/2020/8165296

**Published:** 2020-05-20

**Authors:** Po-Chun Hsieh, Chu-Fang Cheng, Chih-Wei Wu, I-Shiang Tzeng, Chan-Yen Kuo, Pei-Shan Hsu, Chang-Ti Lee, Min-Chien Yu, Chou-Chin Lan

**Affiliations:** ^1^Department of Chinese Medicine, Taipei Tzu Chi Hospital, Buddhist Tzu Chi Medical Foundation, New Taipei City, Taiwan; ^2^School of Post-Baccalaureate Chinese Medicine, Tzu Chi University, Hualien, Taiwan; ^3^Division of Pulmonary Medicine, Taipei Tzu Chi Hospital, Buddhist Tzu Chi Medical Foundation, New Taipei City, Taiwan; ^4^School of Medicine, Tzu-Chi University, Hualien, Taiwan; ^5^Department of Research, Taipei Tzu Chi Hospital, Buddhist Tzu Chi Medical Foundation, New Taipei City, Taiwan

## Abstract

Chronic obstructive pulmonary disease (COPD) is highly prevalent and a major burden on the healthcare system worldwide. It has a severe impact on patients due to poor health-related quality of life (HRQL), dyspnea, and exertional intolerance. Our previous meta-analysis revealed that body acupuncture therapy had adjuvant benefits of improving HRQL in COPD patients undergoing optimal medical treatment. Previous studies indicated that treatment with combinations of acupoints was more effective than single acupoint treatment. The association rule analysis has been widely used to explore relationships in acupoint combination. Therefore, we aimed to investigate the potential core acupoint combination in COPD treatment by mining the association rules from the retrieved randomized control trials (RCTs) of the previous meta-analyses. This study was conducted based on Apriori algorithm-based association rule analysis, which is a popular data mining method available in software R. We extracted acupoints as binary data from the 12 included RCTs for analysis. There were 27 acupoints extracted from 12 RCTs. The top 10 frequently selected acupoints were BL12, BL13, BL20, BL23, BL43, CV17, EXB1, LU5, LU7, and ST36. We investigated 2444 association rules, and the results showed that {ST36, BL12} ≥ {CV17}, {ST36, BL12} ≥ {EXB1}, {CV17, BL12} ≥ {ST36}, and {EXB1, BL12} ≥ {ST36} were the most associated rules in the retrieved RCTs. The acupoint combinations of ST36, BL12, and CV17 and ST36, BL12, and EXB1 could be considered as the core of acupoint combination for further acupuncture treatment of COPD.

## 1. Introduction

Chronic obstructive pulmonary disease (COPD) is highly prevalent and a major burden on the healthcare system worldwide [1]. Besides, patients with COPD often suffer from poor health-related quality of life (HRQL), dyspnea, exercise intolerance, acute exacerbation, and high mortality rate [1]. Although medical efforts have successfully reduced the associated mortality rate in recent years, new problems have been encountered as the Disability-Adjusted of Life Years (DALYs) of COPD rose from level 6 to level 3 [2]. Moreover, increased life expectancy is accompanied with poor HRQL among COPD patients who continue to suffer from symptoms such as dyspnea, chronic cough, and sputum production. Many patients persistently suffer from these symptoms despite optimal pharmacological and nonpharmacological management as per the guidelines of the Global Initiative for Chronic Obstructive Lung Disease (GOLD) [1]. In addition to the prolonged suffering of the patients, the burden of this disease poses significant challenges to the healthcare system [1]. Thus, it is necessary to develop other alternative treatments for COPD.

Acupuncture, a well-practiced therapeutic modality, has attracted attention for its complementary role in alleviating symptoms of certain diseases [3] and for improving the HRQL among COPD patients [4]. Our previous meta-analysis indicated that body acupuncture therapy is an effective adjunctive treatment that improves the HRQL in COPD patients under optimal management [4]. Several possible mechanisms of improving COPD by acupuncture were reported, including anti-inflammatory effects [5], improvement of nutritional state [6, 7], respiratory muscle strength enhancement [6], and improvement of exercise tolerance [8]. It has been widely acknowledged that the selection and combination of acupoints are vital for successful acupuncture treatment. The principles for selecting and combining acupoints are based on the ancient theories of Meridian theory [9] and Biaoben theory [10]. However, there is still no consensus about the standard of acupoints and treatment of COPD.

Recently, data mining methods have been widely used in acupuncture and Chinese medicine. Previous studies based on data mining results provide references for the selection and combination of acupuncture points in treating vascular dementia [11] and Alzheimer's disease [12]. Since the clinical practice of acupuncture is based on the combinations of acupoints, association rule analysis could be a promising and useful method to investigate the underlying rules. However, the study of acupoint combination for the treatment of COPD is lacking. Apriori algorithm is a kind of association rule mining algorithm. It proceeds by identifying the frequent individual item sets in the database [13]. Apriori algorithm-based association rule analysis provides comprehensive and intuitive results to determine association rules which highlight general trends in the database [13].

Therefore, in this study, we aimed to investigate the potential core combination of acupoints for the treatment of COPD, using the Apriori algorithm-based association rule analysis based on our previous systematic review and meta-analysis of randomized controlled trials (RCTs) [4].

## 2. Materials and Methods

### 2.1. Data Sources and Selection Criteria

This study was conducted based on our previous systematic review and meta-analysis [4]. We extracted data on acupoints as binary data from 12 RCT studies (Supplementary Table S1) [4].

We included stable COPD patients diagnosed according to the GOLD guidelines [14] without any exacerbations for at least three months [15]. All the retrieved studies were required to include manual acupuncture or warm acupuncture. Studies with interventions of electroacupuncture, Acu-Transcutaneous Electrical Nerve Stimulation, laser acupuncture, auricular acupuncture, acupressure, sham acupuncture, Chinese herbal medicines, point application, and single moxibustion were excluded.

### 2.2. Risk of Bias Assessment

The methodological quality assessment of the studies was evaluated using the Cochrane RoB 2.0 tool [16]. The tool contained five assessment domains for the risk of bias and led to an overall bias to assess the quality of RCT. The detail of the quality assessment was introduced in our previous study [4].

### 2.3. Data Analysis

We extracted and analyzed the frequency of the acupoints. Definitions about acupoints were as per the World Health Organization standard [17]. In this study, the Apriori algorithm-based association rule analysis and plotting were processed using software R (version 3.4.3). The procedure can be conveniently fitted using the R package “arules,” while the visualizing association rules can be directly fitted using the R package “arulesViz”.

Association rule analysis was one of the main techniques for detecting and extracting useful information from large-scale transaction data [13]. Several studies applied the association rule analysis to investigate hidden structure in medical fields [11, 12, 18]. Basically, an Apriori algorithm-based association rule consists of an antecedent and a consequent, both of which are a list of items. It is important to note that implication here is co-occurrence and not causality. Item set is the list of all the items in the antecedent and the consequent for a given rule.

Four kernel values are involved with association rule analysis, including support, confidence, expected confidence, and lift. Mathematically, support is the fraction of the total number of transactions in which the item set occurs. Technically, confidence is the conditional probability of occurrence of consequent, given the antecedent. Expected confidence indicates the probability of the consequent while consequent was independent of the antecedent. The lift value of an association rule is the ratio of joint probability (of an antecedent and a consequent) and product of their marginal probabilities.

In this study, we conducted the analysis of the top 10 frequently used association rules, and the minimum requirements were determined as support degree ≥20% and confidence ≥80%. Furthermore, we reported the association rules according to descending support and confidence and lift values corresponding to the support of the association rules.

## 3. Results

### 3.1. Study Characteristics and Risk of Bias Assessment

We demonstrated the summary of the retrieved studies and quality assessment with overall bias in Table 1. The full quality assessments are also provided as Supplementary Figure 1. The quality was variable. The primary possible reason is that none of the retrieved studies is patient-blinded study design.

### 3.2. Distribution of the Acupoint

**Figure 1:**
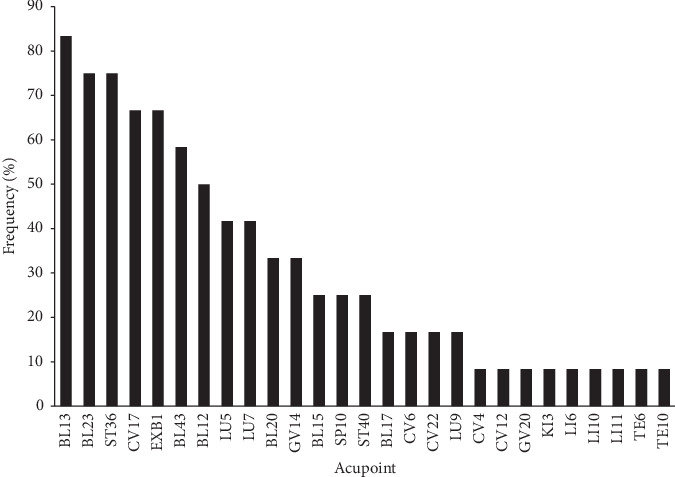
Distribution of acupoints used in the retrieved RCTs.

There were 27 acupoints extracted from the 12 retrieved RCTs from the aforementioned meta-analysis. The distribution details of the acupoint frequency are shown in Figure 1. The top 10 frequently selected acupoints were BL13, BL23, ST36, CV17, EXB1, BL43, BL12, LU5, LU7, and BL20. These acupoints were frequently used in treating respiratory diseases and related symptoms.

### 3.3. Apriori Algorithm-Based Association Rule Analysis for Item Sets of Acupoint Combinations

**Figure 2:**
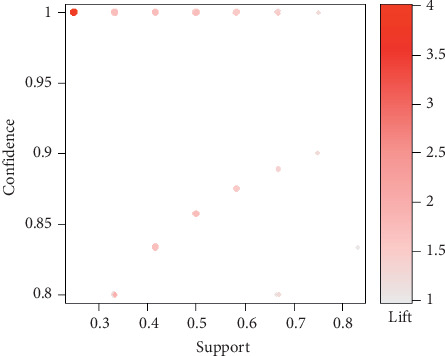
Scatter plot for 2444 rules.

We investigated 2444 association rules based on the integrated acupuncture data (Supplementary Table S1). The association rules were visually presented based on the scatter plot, and the lift of a rule was the ratio of the observed support to that expected if *X* and *Y* were independent (Figure 2). The results demonstrated that all rules had high lift. The most interesting rules (sc-optimal rules) resided on the support/confidence border [13]. The association rules between different individual acupoints were ordered by support. The top 10 Apriori algorithm-based association rules of acupoints are listed in Table 2.

**Figure 3:**
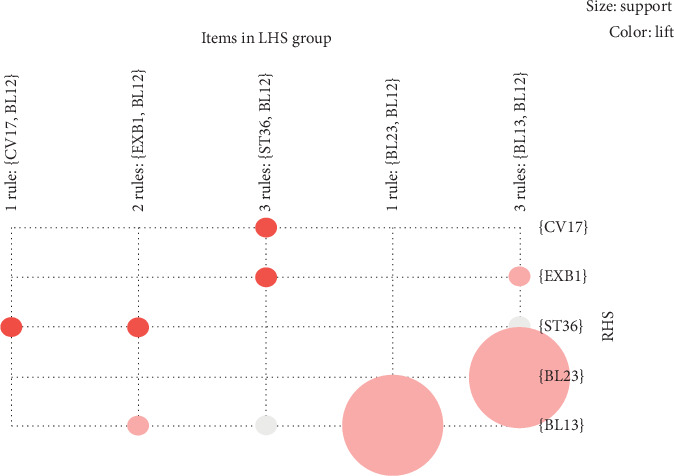
Grouped matrix for 10 association rules.

**Figure 4:**
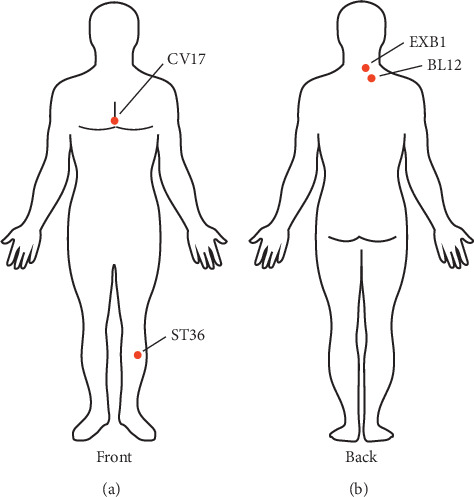
Location of the core acupoints in treating patients with COPD.

With respect to the grouped item sets, we used graph-based visualization by color or size. The features were visually presented based on a grouped matrix of 10 association rules (Figure 3). This plot offered a clear representation of association rules and was appropriate for very small sets of rules to avoid cluttered presentation. Results demonstrated that {ST36, BL12} ≥ {CV17}, {ST36, BL12} ≥ {EXB1}, {CV17, BL12} ≥ {ST36}, and {EXB1, BL12} ≥ {ST36} were interactively selected to reveal the rule's antecedent (LHS) and consequent (RHS) item sets based on the evidence of the grouped matrix for 10 association rules (Figure 4). Compared to Table 2, we found that the interactively selected association rules were consistent for rule numbers 3 ({EXB1} ≥ {ST36}), 4 ({ST36} ≥ {EXB1}), 7 ({CV17} ≥ {ST36}), and 8 ({ST36} ≥ {CV17}).

## 4. Discussion

Our results indicated that ST36, BL12, and CV17 and ST36, BL12, and EXB1 were the core acupoint combinations in treating patients with COPD. Regarding our previous meta-analysis [4], these acupoint combinations played an important role in improving HRQL in patients with COPD under optimal medication. Their results demonstrated evidence-based strategies for acupoint selection in further treatment. To the best of our knowledge, this is the first study to point out the potential core acupoint combination to treat patients with COPD.

The current study confirmed that core acupoint combinations were beneficial for patients with COPD. Anti-inflammatory effects [5], improvement of nutritional state [6, 7], respiratory muscle strength enhancement [6], and improvement of exercise tolerance [8] were reported to be possible mechanisms of improving COPD by acupuncture. Li et al. reported that acupuncture treatment on BL13, BL23, and EXB1 attenuated the inflammatory response (IL-8 and TNF-*α*) by enhancing the expression of mRNA and protein of histone deacetylase 2 (HDAC2) [5]. Poor nutrition in COPD is significantly associated with lower respiratory muscle strength, inefficient ventilation, exercise intolerance, and poor HRQL [19]. Suzuki et al. demonstrated that acupuncture on LU1, LU9, LI18, CV4, CV12, ST36, KI3, GB12, BL13, BL20, and BL23 improved the nutritional state of patients with COPD [6]. The change in body weight, respiratory muscle strength, nutritional hematological examination, and inflammatory biomarkers were significantly improved after acupuncture [6]. Improvement in the gastrointestinal function can result in the improvement of the nutritional status of patients [20]. In a previous review, ST36, PC6, ST37, CV12, and ST25 were the primary acupoints in regulating gastrointestinal function [7]. Maekura et al. demonstrated that acupuncture treatment on LU1, LU9, LI18, CV4, CV12, ST36, KI3, GB12, BL13, BL20, and BL23 improved peak oxygen uptake (V̇O_2peak_), peak minute ventilation (V̇E_peak_), time to the limit of tolerance, and St. George's Respiratory Questionnaire (SGRQ) score of patients with COPD [8].

In clinical practice, acupuncture therapy usually treats patients by employing acupoint combination, rather than a single acupoint. Chen et al. suggested that in cervical spondylosis patients, acupuncture on multiple acupoints resulted in better symptomatic improvement and more decrease in the regional homogeneity in the pain matrix brain area [21]. Zhang et al. reported that in patients with hypertension, acupoint combination of LR3 and KI3 induces better synergistic effects than that of a single acupoint (LR3 or KI3). Resting-state fMRI scan results showed that acupuncture on LR3 and KI3 could activate wider brain areas as compared to the single LR3 or KI3 [22]. Since acupoint combination could enhance effects on the brain area or induce effects on other related areas of the brain compared to a single acupoint; it is important to determine the acupoint combination rather than the single acupoint.

## 5. Conclusions

The acupoint combination of ST36, BL12, and CV17 and ST36, BL12, and EXB1 could be considered the core acupoint combinations for further acupuncture treatments of COPD. Previously, acupuncture was considered a possible but not a reliable treatment, which was deficient in repeated verification. According to our analysis, we recommend the core acupoint combination for further basic mechanism research studies, clinical trials, and treatment strategies.

### 5.1. Limitation

Although we suggested the core of acupoint combination, our study had a few limitations. First, there are many factors that could influence the effects of acupuncture, including depth of needling, manipulation method of acupuncture, time of retaining the needle, treatment frequency, and treatment course. However, these factors were not addressed in this analysis. Second, other acupuncture systems, including scalp acupuncture, auricular acupuncture, Tung's Acupuncture, etc., and their roles were not addressed in this study. Third, the mechanisms of acupoint combination are still unclear. Therefore, further basic and clinical studies are necessary for a thorough evaluation.

## Figures and Tables

**Table 1:** Summary of the retrieved studies.

Author	Year	Study design	Diagnosis	Acupoints	Overall bias (Rob 2.0)
Jia	2004	RCT	COPD stage II or III	BL13, BL43, LU9, KI3, BL23, ST36, LU7, LU5, CV17, EXB1	High
Deering et al.	2011	RCT	COPD	LI11, LI10, TE10, TE6, L5, L7	High
Fan et al.	2011	RCT	COPD	EXB1, BL13, BL12, BL43, BL15, CV17, CV22, BL20, BL23, ST36	Some
Gao et al.	2011	RCT	COPD	EXB1, BL13, ST36, BL12, GV14, BL43, BL15, CV17, CV22, BL20, BL23	High
Xie et al.	2014	RCT	COPD	ST36, BL13, EXB1, BL43, BL15, GV14, BL12	Some
Yu	2014	RCT	COPD stage II or III	BL13, BL12, CV17, EXB1, BL43, BL23, ST36, LU7, LU5, ST40, SP10	High
Lee et al.	2015	RCT	COPD	GV14, BL13, BL20, BL23, BL17	Low
Liu et al.	2015	RCT	COPD stage III or IV	BL13, BL23, CV6, CV4, EXB1, CV17, ST36	High
Yang et al.	2016	RCT	COPD	GV14, BL13, BL20, BL23, BL17	Low
Chu	2017	RCT	COPD	ST36, BL23, BL43, EXB1, CV17, BL12, BL13, SP10, ST40, LU5, LU7	High
Lee	2017	RCT	COPD	BL13, BL12, CV17, EXB1, BL43, BL23, ST36, LU5, ST40, SP10	Some
Shi et al.	2017	RCT	COPD	CV17, CV12, CV6, GV20, LI6, LU7, LU9, ST36	High

RCT: randomized control trial; COPD: chronic obstructive pulmonary disease; Rob 2.0: RoB 2.0 tool (revised tool for risk of bias in randomized trials).

**Table 2:** Top 10 Apriori algorithm-based association rules of acupoints.

No.	Association rules	Support	Confidence	Expected confidence	Lift
1	{BL23} ≥ {BL13}	0.7500000	1.0000000	0.833333	1.200000
2	{BL13} ≥ {BL23}	0.7500000	0.9000000	0.750000	1.200000
3	{EXB1} ≥ {ST36}	0.6666667	1.0000000	0.750000	1.333333
4	{ST36} ≥ {EXB1}	0.6666667	0.8888889	0.666667	1.333333
5	{EXB1} ≥ {BL13}	0.6666667	1.0000000	0.833333	1.200000
6	{BL13} ≥ {EXB1}	0.6666667	0.8000000	0.666667	1.200000
7	{CV17} ≥ {ST36}	0.6666667	1.0000000	0.750000	1.333333
8	{ST36} ≥ {CV17}	0.6666667	0.8888889	0.666667	1.333333
9	{ST36} ≥ {BL13}	0.6666667	0.8888889	0.833333	1.066667
10	{BL13} ≥ {ST36}	0.6666667	0.8000000	0.750000	1.066667

## Data Availability

The original data used to support the findings of this study are included within the article.

## Supplementary Materials

Supplementary Materials Supplementary Figure 1: risk of bias assessment of the retrieved RCTs. Supplementary Table 1: binary data of acupoints extracted from 12 RCT studies.
