# Supplementary material for: Combination of Acupoints in Treating Patients with Chronic Obstructive Pulmonary Disease: An Apriori Algorithm-Based Association Rule Analysis
Source: Evid Based Complement Alternat Med. 2020 May 20;2020:8165296. doi: 10.1155/2020/8165296 (PMC7256717; doi:10.1155/2020/8165296)
Supplement: Supplementary Materials — Supplementary Figure 1: risk of bias assessment of the retrieved RCTs. Supplementary Table 1: binary data of acupoints extracted from 12 RCT studies. [file 8165296.f1.pdf]

Supplementary Figure 1. Risk of bias assessment of the retrieved RCTs.

A

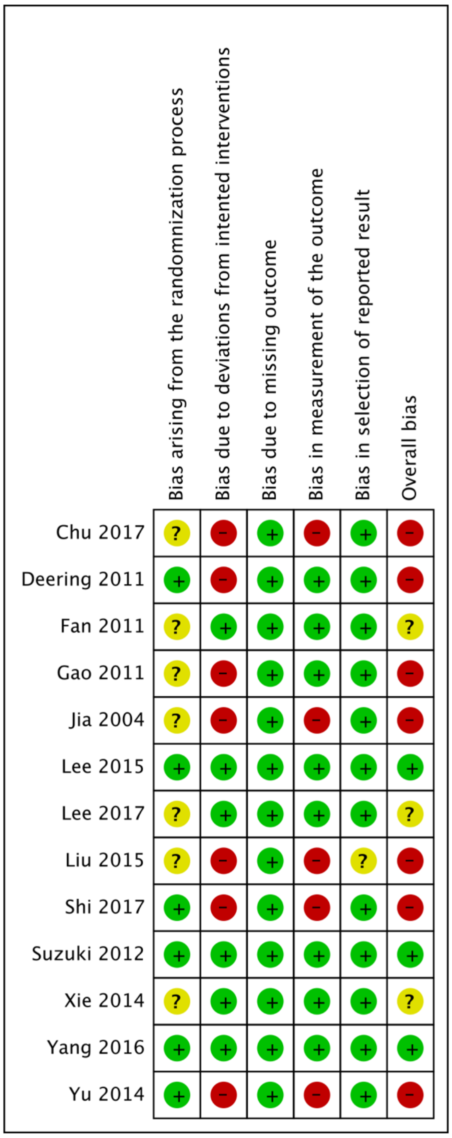

B

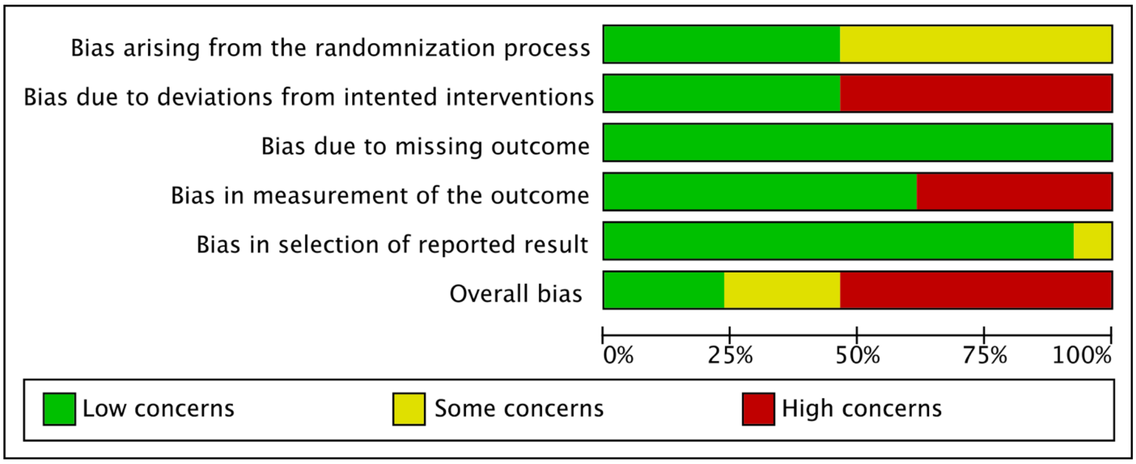

Supplementary Table 1. Binary data of acupoints extracted from 12 RCT studies

Study	BL12	BL13	BL15	BL17	BL20	BL23	BL43	CV12	CV17	CV22	CV4	CV6	EXB1	GV14	GV20	KI3	LI10	LI11	LI6	LU5	LU7	LU9	SP10	ST36	ST40	TE10	TE6
Jia (2004)	0	1	0	0	0	1	1	0	1	0	0	0	1	0	0	1	0	0	0	1	1	1	0	1	0	0	0
Deering et al. (2011)	0	0	0	0	0	0	0	0	0	0	0	0	0	0	0	0	1	1	0	1	1	0	0	0	0	1	1
Fan et al. (2011)	1	1	1	0	1	1	1	0	1	1	0	0	1	0	0	0	0	0	0	0	0	0	0	1	0	0	0
Gao et al. (2011)	1	1	1	0	1	1	1	0	1	1	0	0	1	1	0	0	0	0	0	0	0	0	0	1	0	0	0
Xie et al. (2014)	1	1	1	0	0	0	1	0	0	0	0	0	1	1	0	0	0	0	0	0	0	0	0	1	0	0	0
Yu (2014)	1	1	0	0	0	1	1	0	1	0	0	0	1	0	0	0	0	0	0	1	1	0	1	1	1	0	0
Lee et al. (2015)	0	1	0	1	1	1	0	0	0	0	0	0	0	1	0	0	0	0	0	0	0	0	0	0	0	0	0
Liu et al. (2015)	0	1	0	0	0	1	0	0	1	0	1	1	1	0	0	0	0	0	0	0	0	0	0	1	0	0	0
Yang et al. (2016)	0	1	0	1	1	1	0	0	0	0	0	0	0	1	0	0	0	0	0	0	0	0	0	0	0	0	0
Chu (2017)	1	1	0	0	0	1	1	0	1	0	0	0	1	0	0	0	0	0	0	1	1	0	1	1	1	0	0
Lee (2017)	1	1	0	0	0	1	1	0	1	0	0	0	1	0	0	0	0	0	0	1	0	0	1	1	1	0	0
Shi et al. (2017)	0	0	0	0	0	0	0	1	1	0	0	1	0	0	1	0	0	0	1	0	1	1	0	1	0	0	0
